# Relevance of the Mention of Antioxidant Properties in Yogurt Labels: *In Vitro* Evaluation and Chromatographic Analysis

**DOI:** 10.3390/antiox2020062

**Published:** 2013-06-18

**Authors:** Eliana Pereira, Lillian Barros, Isabel C. F. R. Ferreira

**Affiliations:** CIMO-ESA, Instituto Politécnico de Bragança, Campus de Santa Apolónia, Apartado 1172, Bragança 5301-855, Portugal; E-Mails: eliana.pereira_19@hotmail.com (E.P.); lillian@ipb.pt (L.B.)

**Keywords:** yogurt, fruits, antioxidant activity, sugars, tocopherols

## Abstract

The purpose of the inclusion of fruit (natural additives) in yogurt aims to increase its antioxidant activity and functionality. Herein, a comparative study of the antioxidant potential of yogurts with pieces of various fruits was performed, including yogurts with mention of antioxidant properties in the label. Free radicals scavenging activity, reducing power and inhibition of lipid peroxidation were evaluated by *in vitro* assays, as were the contents in antioxidants such as phenolics, flavonoids, sugars and tocopherols. After analyzing thirteen yogurts containing fruit pieces and a natural one (control), the most interesting were yogurts with pieces of berries (for phenolics, flavonoids and 2,2-dipheny-1-picrylhydrazyl (DPPH) scavenging activity), pineapple (for reducing power), blackberry (for β-carotene bleaching inhibition), blackberry “antioxidant” (for tocopherols) and cherry (for sugars). The mention of “antioxidant” in the label was relevant for tocopherols, sugars, DPPH scavenging activity and reducing power. No synergisms were observed in yogurts prepared with pieces of different fruits. Nevertheless, the addition of fruit pieces to yogurt was favorable for antioxidant content, increasing the protection of the consumer against diseases related to oxidative stress.

## 1. Introduction

Yogurt is formed during the slow lactic fermentation of lactose from milk by thermophilic lactic acid bacteria, and is one of the most popular fermented foods and traditionally consumed in many countries [1,2]. It is widely consumed as functional food because of the good taste and nutritional properties (rich in potassium, calcium, protein and vitamins), being an excellent vehicle to deliver probiotics to consumers [3].

Yogurt is also produced and consumed in flavored and supplemented forms. The flavoring and pigment can be done by addition of natural ingredients or by addition of synthetic flavor compounds, adding fruit juices or fruit pulp. Many of these fruits are known as very good sources of anthocyanins, which display a wide range of biological activities including antioxidant, anti-inflammatory, antimicrobial and anti-carcinogenic activities [4,5,6].

This enrichment with various physiologically active ingredients provide specific health benefits beyond basic nutrition, such as omega-3 fatty acids, phytosterols antioxidant food ingredients, vegetable fibers and aqueous extracts. The reason for incorporating ingredients with antioxidant activity is to enhance the functionality and activity of these foodstuffs and in this way to improve consumer’s protection against pathologies related to free radicals [7]. Regular consumption of yogurt seems to be beneficial in the strengthening of the immune system, improvement in lactose digestion, blood glucose management and in the reduction of constipation, diarrhea, cancer prevention, inflammatory bowel disease and allergies [3].

Diverse studies have shown that free radicals present in the human organism cause oxidative damage to different molecules, such as lipids, proteins and nucleic acids and thus are involved in the initiation phase of some degenerative diseases. In this way, the antioxidant compounds are capable of neutralizing free radicals and may play an important role in the prevention of these diseases. Fruits contain different antioxidant compounds, such as vitamins and carotenoids, whose activities have been established in recent years. However, these compounds are not the only ones contributing to the antioxidant activity of fruits; there is also the presence of polyphenols, such as flavonoids (anthocyanins, flavonols, catechins, flavones and flavanones) that bring different beneficial effects [8]. Nevertheless, the health effects of polyphenols depend on the amount consumed and their bioavailability [6].

Despite some published studies on yogurt functional properties [9,10], comparative reports on antioxidant activity of different samples available in the market are scarce. Therefore, in the present work, thirteen yogurt samples with fruit pieces were studied and compared with a natural one (control sample). The selection was made in order to include fruits known as powerful antioxidant sources such as berries, blackberry, cherry, mango, peach, pineapple, plum, raspberry and strawberry-kiwi [11,12,13,14,15,16]. Furthermore, the relevance of the information "antioxidant" on the yogurt label was evaluated, comparing samples having this mention with samples with the same fruit pieces but without the “antioxidant” mention in the label.

## 2. Experimental Section

### 2.1. Samples

Fourteen yogurts with pieces of fruits were obtained from local supermarket. The selection was performed taking into account: (i) presence of fruits with known antioxidant activity; (ii) diversity of fruits; and (iii) mention of “antioxidant” in the label. Information about the samples is provided in Table 1. All the samples were lyophilized (FreeZone 4.5 model 7750031, Labconco, KA, USA), reduced to a fine dried powder (20 mesh) and kept at −20 °C until further analysis.

**Table 1:** Composition of the studied yogurts according to the label.

Designation	Composition
Berries	Pasteurized skimmed milk, fruit pieces 11.6%, strawberries 5.6%, blueberries 2.2%, blackberries 1.9%, raspberries 1.9%, skimmed milk powder, lactic ferments, sweeteners (aspartame and acesulfame K), flavorings, colors (carmins), fruit preservative (E-202). Contains a source of phenylalanine.
Berries “Antioxidant” ^a^	Pasteurized skimmed milk, pasteurized milk, sugar, blackberry, strawberry, raspberry (9%), skim milk powder, milk proteins, dextrose, grape natural extract (0.07%), flavorings (blackberry, strawberry and raspberry), lactic ferments, fruit preservative (potassium sorbate).
Blackberry	Pasteurized milk, reconstituted milk, blackberry pieces 10%, sugar 9.4%, glucose-fructose syrup, skimmed milk powder and/or milk proteins, modified starch, thickeners (pectin, guar gum), flavorings, colors (anthocyanins), lactic ferments.
Blackberry “antioxidant” ^a^	Pasteurized skimmed milk, pasteurized milk, sugar, blackberry 9%, skim milk powder, milk proteins, dextrose, grape natural extract 0.07%, flavoring (blackberry), lactic ferments, fruit preservative (potassium sorbate).
Cherry	Partially skimmed-milk, fruits (cherry 10.2%), sugar 8.4%, milk proteins, corn modified starch, thickeners (guar gum, pectin), acidifying substances (citric acid, calcium citrate, sodium citrate), flavorings, colors (concentrated elderberry juice), fruit preservative (potassium sorbate), lactic ferments.
Cherry burlat “antioxidant” ^a^	Pasteurized skimmed milk, pasteurized milk, sugar, burlat cherry 9%, skim milk powder, milk proteins, dextrose, grape natural extract 0.07%, flavorings (cherry), lactic ferments, fruit preservative (potassium sorbate).
Cherry griotte “antioxidant” ^a^	Pasteurized skimmed milk, pasteurized milk, sugar, griotte cherry 9%, skim milk powder, milk proteins, dextrose, grape natural extract 0.07%, flavorings (cherry), lactic ferments, fruit preservative (potassium sorbate).
Mango	Whole milk, mango 10%, sugar 9.2%, milk proteins, wheat dextrin (food fibers), modified cornstarch, thickeners (xanthan gum, guar flour), flavorings, color (pepper extract), lactic ferments including bifidobacteria.
Peach	Pasteurized milk, pasteurized milk reconstituted, peach pieces 13%, sugar, milk proteins, acacia gum, flavorings (peach), colors (E-160a and E-160c), bifidobacteria and active lactic ferments, fruit preservative (E-202).
Pineapple	Pasteurized skimmed milk, pulp and pineapple pieces 11.6%, skimmed milk powder, acacia gum, sweeteners (aspartame and acesulfame K), flavorings (pineapple), bifidobacteria and active lactic ferments, fruit preservative (E-202). Contains a source of phenylalanine.
Plum	Whole milk, plums 10% (rehydrated plums and plums puree), sugar 5.7%, wheat glucose-fructose syrup, milk proteins, wheat dextrin (food fibers), thickeners (carrageenan, pectin, xanthan gum), lactic ferments including bifidobacteria, fruit preservative (potassium sorbate), flavorings.
Raspberry	Pasteurized milk, reconstituted milk, raspberry pieces 9.4%, sugar 9.2%, glucose-fructose syrup, skimmed milk powder and/or milk proteins, modified starch, thickeners (pectin, guar gum), flavorings, colors (anthocyanins), lactic ferments.
Strawberry-Kiwi	Pasteurized milk, skimmed-milk powder, strawberry pieces 6%, kiwi pulp and pieces 5.6%, acacia gum, flavorings, sweeteners (aspartame and acesulfame K), bifidobacteria and active lactic ferments, colors (carmins), fruit preservative (E-202). Contains a source of phenylalanine.
Natural	Partially skimmed milk, milk proteins, selected lactic ferments.

^a^ These samples contain the mention “antioxidant” in the label.

### 2.2. Standards and Reagents

Acetonitrile 99.9%, *n*-hexane 95% and ethyl acetate 99.8% were of HPLC grade from Fisher Scientific (Lisbon, Portugal). Potassium ferricyanide, sodium hydroxide, aluminum chloride, sodium nitrite, trichloroacetic acid, tocopherols (α-, β-, γ-, and δ-isoforms), sugars (d(−)-fructose, d(+)-glucose, d(+)-galactose, d(+)-sucrose, lactose 1-hydrate, d(+)-maltose and d(−)-raffinose pentahydrate), trolox (6-hydroxy-2,5,7,8-tetramethylchroman-2-carboxylic acid), gallic acid and (+)-catechin standards were purchased from Sigma (St. Louis, MO, USA). Racemic tocol, 50 mg/mL, was purchased from Matreya (Pleasant Gap, PA, USA). 2,2-Diphenyl-1-picrylhydrazyl (DPPH) was obtained from Alfa Aesar (Ward Hill, MA, USA). All other chemicals and solvents were of analytical grade and purchased from common sources. Water was treated in a Milli-Q water purification system (TGI Pure Water Systems, USA).

### 2.3. Apparatus

HPLC equipment consisted of an integrated system with a pump (Knauer, Smartline system 1000, Berlin, Germany), degasser system (Smartline manager 5000), auto-sampler (AS-2057 Jasco), RI detector (Knauer Smartline 2300), fluorescence detector (FP-2020; Jasco, Easton, MD, USA), Clarity 2.4 Software (DataApex) and an oven (7971 R Grace oven). Rotary evaporator (Büchi R-210; Flawil, Switzerland), ELX800 Microplate Reader (Bio-Tek, Bedfordshire, UK) and a spectrophotometer (AnalytikJena, Jena, Germany) were also used.

### 2.4. *In Vitro* Evaluation of Antioxidant Activity

#### 2.4.1. Extraction Procedure and General Information

The lyophilized sample (~4 g) was stirred with 50 mL of ethanol:water (80:20 v/v) at 25 °C at 150 rpm for 1 h and filtered through Whatman No. 4 paper. The residue was then extracted with one additional 50 mL portion of ethanol:water (80:20 v/v). The combined extracts were evaporated under reduced pressure, re-dissolved in ethanol:water (80:20 v/v) at 200 mg/mL, and stored at 4 °C for further use. Successive dilutions were made from the stock solution and submitted to *in vitro* assays already described by the authors [17] to evaluate the antioxidant activity of the samples. The sample concentrations (ranging from 200 to 0.625 mg/mL extract solution) providing 50% of antioxidant activity or 0.5 of absorbance (EC_50_) were calculated from the graphs of antioxidant activity percentages (DPPH, β-carotene/linoleate and TBARS assays) or absorbance at 690 nm (reducing power assay) against sample concentrations. Trolox was used as standard (in a range of 250–2 μg/mL).

#### 2.4.2. DPPH Radical-Scavenging Activity

This methodology was performed using a Microplate Reader. The reaction mixture in each one of the 96-wells consisted of one of the different concentrations of the extracts (ranging from 200–0.625 mg/mL; 30 μL) and methanolic solution (270 μL) containing DPPH radicals (6 × 10^−5^ mol/L). The mixture was left to stand for 60 min in the dark. The reduction of the DPPH radical was determined by measuring the absorption at 515 nm. The radical scavenging activity (RSA) was calculated as a percentage of DPPH discoloration using the equation:

% RSA = [(*A*_DPPH_ − *A*_S_)/*A*_DPPH_] × 100 (1)

where *A*_S_ is the absorbance of the solution when the sample extract has been added at a particular level, and *A*_DPPH_ is the absorbance of the DPPH solution [17].

#### 2.4.3. Reducing Power

The different concentrations of the extracts (ranging from 200 to 0.625 mg/mL; 0.5 mL) were mixed with sodium phosphate buffer (200 mmol/L, pH 6.6, 0.5 mL) and potassium ferricyanide (1% w/v, 0.5 mL). For each concentration, the mixture was incubated at 50 °C for 20 min, and trichloroacetic acid (10% w/v, 0.5 mL) was added. The mixture (0.8 mL) was poured in the 48-wells, as also deionized water (0.8 mL) and ferric chloride (0.1% w/v, 0.16 mL), and the absorbance was measured at 690 nm in the the Microplate Reader [17].

#### 2.4.4. Inhibition of β-Carotene Bleaching

β-carotene (2 mg) was dissolved in chloroform (10 mL) and 2 mL of this solution were pipetted into a round-bottom flask. After the chloroform was removed at 40 °C under vacuum, linoleic acid (40 mg), Tween 80 emulsifier (400 mg), and distilled water (100 mL) were added to the flask with vigorous shaking. Aliquots (4.8 mL) of this emulsion were transferred into different test tubes containing different concentrations of the extracts (ranging from 200 to 0.625 mg/mL; 0.2 mL). The tubes were shaken and incubated at 50 °C in a water bath. As soon as the emulsion was added to each tube, the zero time absorbance was measured at 470 nm in a spectrophotometer. β-Carotene bleaching inhibition was calculated using the following equation [17]:

(Absorbance after 2 h of assay/initial Absorbance) × 100
(2)

### 2.5. Antioxidants Determination

#### 2.5.1. Phenolics

The extract solution (50 mg/mL; 1 mL) was mixed with Folin-Ciocalteu reagent (5 mL, previously diluted with water 1:10, v/v) and sodium carbonate (75 g/L, 4 mL). The tubes were vortex mixed for 15 s and allowed to stand for 30 min at 40 °C for color development. Absorbance was then measured at 765 nm in a spectrophotometer. Gallic acid was used to obtain the standard curve (9.4 × 10^−3^–1.5 × 10^−1^ mg/mL), and the results were expressed as mg of gallic acid equivalents (GAE) per g of extract [17].

#### 2.5.2. Flavonoids

The extract solution (200 mg/mL; 0.5 mL) was mixed with distilled water (2 mL) and subsequently with NaNO_2_ solution (5%, 0.15 mL). After 6 min, AlCl_3_ solution (10%, 0.15 mL) was added and allowed rest for further 6 min. A NaOH solution (4%, 2 mL) was added to the mixture, and distilled water was immediately added to bring the final volume to 5 mL. The mixture was then properly mixed and allowed to stand for 15 min. The absorbance of a pink color was measured at 510 nm in a spectrophotometer. (+)-Catechin was used to calculate the standard curve (4.5 × 10^−3^–2.9 × 10^−1^ mg/mL) and the results were expressed as mg of (+)-catechin equivalents (CE) per g of extract [17].

#### 2.5.3. Sugars

Free sugars were determined by high performance liquid chromatography coupled with a refraction index detector (HPLC-RI), after an extraction procedure previously described by the authors [18], using raffinose as internal standard (IS). The chromatographic separation was achieved with a Eurospher 100-5 NH_2 _column (4.6 × 250 mm, 5 mm, Knauer, Berlin, Germany) operating at 30 °C. The elution procedure was isocratic and the mobile phase was acetonitrile/deionized water, 70:30 (v/v) at a flow rate of 1 mL/min. The compounds were identified by chromatographic comparisons with authentic standards. Quantification was performed using the internal standard method and analyzed using Clarity 2.4 Software. Sugar contents were further expressed in g per 100 g of fresh weight (*fw*).

#### 2.5.4. Tocopherols

Tocopherols were determined by HPLC-fluorescence, after an extraction procedure previously described by the authors [18], using tocol as IS. The analysis was carried out in the HPLC system described above connected to a fluorescence detector programmed for excitation at 290 nm and emission at 330 nm. The chromatographic separation was achieved with a Polyamide II normal-phase column (250 × 4.6 mm; YMC Waters) operating at 30 °C. The elution procedure was isocratic and the mobile phase used was a mixture of *n*-hexane and ethyl acetate (70:30, v/v) at a flow rate of 1 mL/min. The compounds were identified by chromatographic comparisons with authentic standards. Quantification was performed, using the internal standard method and analyzed using Clarity 2.4 Software. Tocopherol contents were further expressed in μg per 100 g of fresh weight (*fw*).

### 2.6. Statistical Analysis

For each one of the yogurts, three samples were used and all the assays were carried out in triplicate. The results are expressed as mean values and standard deviation (SD). The results were analyzed using one-way analysis of variance (ANOVA) followed by Tukey’s HSD Test with α = 0.05. This treatment was carried out using SPSS version 18.0 program (IBM Corporation, New York, NY, USA).

## 3. Results and Discussion

The results of the *in vitro* antioxidant activity, phenolics and flavonoids content of the studied yogurts are shown in Table 2. The highest concentration of phenolics and flavonoids was found in berry yogurt (6.91 mg GAE/g extract and 2.98 CE mg/g extract, respectively), while the lowest values were obtained in mango yogurt (1.07 mg GAE/g extract and 0.01 mg CE/g extract, respectively). Flavonoids were not detected in peach and natural yogurts. Berries and cherry Burlat “antioxidant” yogurts gave the highest DPPH scavenging activity (no significant, *p* < 0.05 statistical differences between EC_50_ values: 11.95 and 11.35 mg/mL), pineapple yogurt revealed the highest reducing power (1.74 mg/mL), and blackberry, cherry, cherry Griotte “antioxidant” and raspberry gave the highest β-carotene bleaching inhibition capacity without significant statistical differences (Table 2).

**Table 2:** Antioxidants content and *in vitro* antioxidant properties of the studied yogurts (mean ± SD).

	Phenolics (mg GAE/g extract)	Flavonoids (mg CE/g extract)	DPPH scavenging activity (EC_50_; mg/mL)	Reducing power (EC_50_; mg/mL)	β-carotene bleaching inhibition (EC_50_; mg/mL)
Berries	6.91 ± 0.45 ^a^	2.98 ± 0.69 ^a^	11.95 ± 0.44 ^h^	4.71 ± 0.09 ^j^	53.90 ± 4.14 ^de^
Berries “*antioxidant*” ^a^	3.32 ± 0.05 ^e^	0.13 ± 0.02 ^de^	28.34 ± 1.60 ^fg^	8.56 ± 0.39 ^h^	71.09 ± 8.73 ^d^
Blackberry	2.69 ± 0.09 ^f^	0.76 ± 0.51 ^cd^	43.61 ± 6.07 ^de^	17.24 ± 0.09 ^c^	1.37 ± 0.10 ^g^
Blackberry “*antioxidant*” ^a^	4.15 ± 0.23 ^c^	0.39 ± 0.03 ^de^	19.09 ± 0.97 ^gh^	7.77 ± 0.48 ^i^	38.57 ± 3.15 ^ef^
Cherry	3.49 ± 0.03 ^de^	0.35 ± 0.11 ^de^	36.07 ± 1.52 ^ef^	11.12 ± 0.06 ^e^	8.83 ± 0.79 ^g^
Cherry Burlat “*antioxidant*” ^a^	3.69 ± 0.09 ^d^	1.42 ± 1.08 ^bc^	11.35 ± 0.79 ^h^	8.43 ± 0.37 ^h^	49.89 ± 1.15 ^e^
Cherry Griotte “*antioxidant*” ^a^	3.41 ± 3.38 ^de^	1.71 ± 0.95 ^b^	31.11 ± 1.80 ^f^	10.39 ± 0.04 ^f^	12.61 ± 6.10 ^g^
Mango	1.07 ± 0.04 ^i^	0.01 ± 0.18 ^e^	42.47 ± 5.00 ^de^	23.61 ± 0.26 ^b^	34.48 ± 0.10 ^ef^
Peach	1.42 ± 0.02 ^h^	nd	37.22 ± 4.68 ^def^	13.59 ± 0.57 ^d^	20.67 ± 1.56 ^fg^
Pineapple	6.90 ± 0.31 ^a^	0.11 ± 0.22 ^de^	44.21 ± 15.16 ^de^	1.74 ± 0.03 ^l^	289.01 ± 3.56 ^a^
Plum	2.18 ± 0.00 ^g^	0.27 ± 0.15 ^de^	82.01 ± 7.04 ^a^	9.58 ± 0.36 ^g^	226.57 ± 12.97 ^b^
Raspberry	2.38 ± 0.04 ^fg^	0.44 ± 0.12 ^de^	57.03 ± 3.39 ^b^	26.68 ± 0.15 ^a^	9.50 ± 0.45 ^g^
Strawberry-kiwi	4.12 ± 0.16 ^c^	0.48 ± 0.23 ^de^	45.45 ± 5.02 ^cd^	7.40 ± 0.19 ^i^	52.26 ± 0.07 ^de^
Natural (control)	5.14 ± 0.16 ^b^	nd	54.26 ± 6.41 ^bc^	2.74 ± 0.07 ^k^	175.40 ± 20.06 ^c^

^a^ These samples contain the mention “antioxidant” in the label. nd—not detected; GAE—gallic acid equivalents; CE—catechin equivalents. EC_50_: Extract concentration corresponding to 50% of antioxidant activity or 0.5 of absorbance for the reducing power assay. In each column different letters (from a to l) mean statistical significant differences (“a” being the highest value) differences between species (*p* < 0.05).

Disparity among the samples that gave the highest antioxidant activity in each one of the used assays is an evidence of the different mechanisms involved and compounds responsible for those mechanisms [19,20,21]. The possibility of pro-oxidation effects [22], for example in pineapple yogurt, should not be discarded, since this sample showed the highest levels of phenolics but not the highest antioxidant properties.

**Figure 1:**
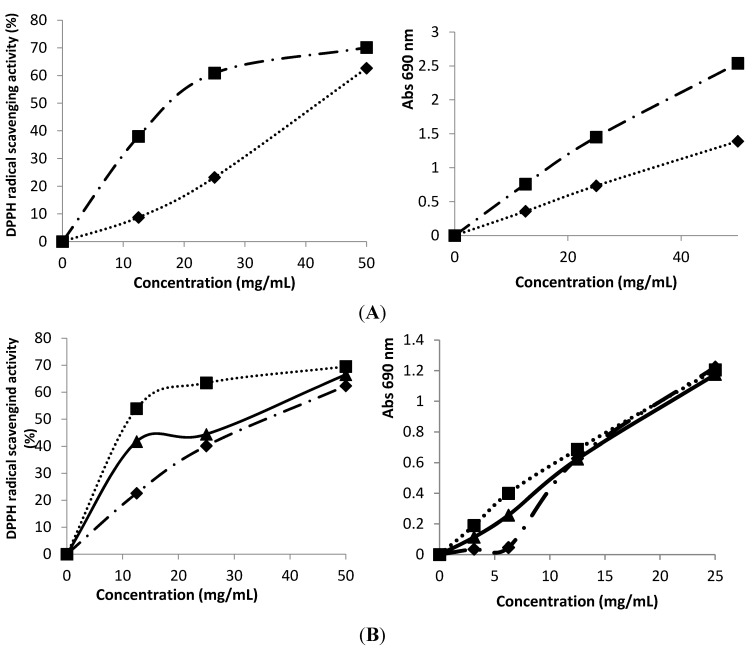
Comparison of 2,2-Diphenyl-1-picrylhydrazyl (DPPH) scavenging activity and reducing power between (**A**) 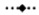 blackberry and 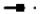 blackberry “antioxidant” yogurts; (**B**) 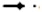 cherry and cherry “antioxidant” 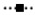 Burlat and 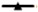 Griotte.

The relevance of “antioxidant” mention in the label was evident in some cases such as blackberries “antioxidant” yogurt that revealed higher phenolic content, DPPH scavenging activity and reducing power than blackberry yogurt (Figure 1A; Table 2), and cherry Burlat and Griotte “antioxidant” yogurt that gave also higher DPPH scavenging activity and reducing power than cherry yogurt (Figure 1B). The same was not observed for berries samples, where the yogurt with “antioxidant” mention revealed lower phenolic content and antioxidant activity. The natural yogurt was used as a control sample, and when compared to the study presented by Ye *et al.* [23], the results were similar for the DPPH scavenging activity (48.704–42.857 mg/mL), but higher for reducing power (21.123–16.172 mg/mL) and lower for β-carotene bleaching inhibition (29.284–19.032 mg/mL).

**Table 3:** Sugars composition of the studied yogurts obtained by chromatographic analysis (mean ± SD) and comparison with the information about total sugars available in the label.

	Fructose	Glucose	Galactose	Sucrose	Lactose	Maltose	Total sugars	Total soluble sugars (label)
Berries	0.49 ± 0.02 ^ef^	0.42 ± 0.04 ^e^	1.01± 0.06 ^a^	0.41 ± 0.05 ^g^	5.40 ± 0.39 ^a^	nd	7.73 ± 0.48 ^efg^	5.5
Berries “*antioxidant*” ^a^	0.39 ± 0.11 ^f^	0.75 ± 0.22 ^d^	0.50 ± 0.13 ^cd^	5.69 ± 1.83 ^de^	3.08 ± 0.69 ^ef^	nd	10.41 ± 2.98 ^de^	14.4
Blackberry	1.22 ± 0.12 ^b^	1.38 ± 0.11 ^c^	0.31 ± 0.09 ^e^	4.88 ± 0.25 ^e^	2.25 ± 0.11 ^fg^	nd	10.04 ± 0.68 ^de^	14.9
Blackberry “*antioxidant*”^ a^	0.75 ± 0.13 ^cd^	1.23 ± 0.21 ^c^	0.66 ± 0.10 ^bc^	8.09 ± 1.53 ^abc^	4.17± 0.67 ^bcd^	nd	14.90 ± 2.64 ^ab^	14.4
Cherry	2.02 ± 0.24 ^a^	1.15 ± 0.17 ^c^	0.65 ± 0.05 ^bc^	8.30 ± 0.63 ^ab^	4.51 ± 0.00 ^abc^	nd	16.63 ± 1.09 ^a^	13.5
Cherry Burlat “*antioxidant*” ^a^	0.92 ± 0.02 ^c^	1.70 ± 0.04 ^b^	0.61 ± 0.01 ^bcd^	6.52 ± 0.59 ^cde^	3.78 ± 0.25 ^cde^	nd	13.53 ± 0.91 ^bc^	14.7
Cherry Griotte “*antioxidant*” ^a^	1.27 ± 0.04 ^b^	2.12 ± 0.14 ^a^	0.61 ± 0.05 ^bcd^	6.71 ± 0.35 ^bcd^	3.98 ± 0.24 ^bcde^	nd	14.69 ± 0.72 ^ab^	14.7
Mango	0.63 ± 0.02 ^de^	0.45 ± 0.02 ^e^	0.68 ± 0.02 ^b^	9.11 ± 0.01 ^a^	3.71 ± 0.02 ^cde^	nd	14.58 ± 0.07 ^ab^	14.1
Peach	0.45 ± 0.08 ^ef^	0.50 ± 0.09 ^de^	0.59 ± 0.09 ^bcd^	6.17 ± 1.01 ^de^	3.12 ± 0.54 ^ef^	nd	10.83 ± 1.82 ^cd^	12.4
Pineapple	0.44 ± 0.06 ^ef^	0.57 ± 0.07 ^de^	0.97 ± 0.14 ^a^	0.26 ± 0.03 ^g^	4.73 ± 0.60 ^ab^	nd	6.97 ± 0.90 ^fg^	7.3
Plum	0.84 ± 0.05 ^cd^	2.18 ± 0.23 ^a^	nd	2.24 ± 0.20 ^f^	1.61 ± 0.13 ^g^	1.13 ± 0.01	9.04 ± 0.15 ^def^	13.7
Raspberry	0.82 ± 0.15 ^cd^	1.36 ± 0.20 ^c^	0.46 ± 0.09 ^de^	8.27 ± 1.27 ^ab^	3.31 ± 0.60 ^de^	nd	14.22 ± 2.31 ^ab^	14.9
Strawberry-kiwi	0.43 ± 0.06 ^ef^	0.41 ± 0.07 ^e^	0.95 ± 0.11 ^a^	0.15 ± 0.06 ^g^	4.56 ± 0.66 ^abc^	nd	6.50 ± 0.95 ^fg^	7.1
Natural (control)	nd	nd	0.91 ± 0.05 ^a^	nd	3.80 ± 0.25 ^cde^	nd	4.71 ± 0.30 ^g^	na

^a^ These samples contain the mention “antioxidant” in the label. nd—not detected; na—not available. The results are expressed in g/100 g fresh weight. In each column different letters (from a to g) mean statistical significant differences (“a” being the highest value) between species (*p* < 0.05).

Some sugars have reducing properties being able to act as antioxidants [24,25,26], such is the case of fructose, glucose, galactose, maltose and lactose, found in some of the studied yogurts (Table 3).

**Figure 2:**
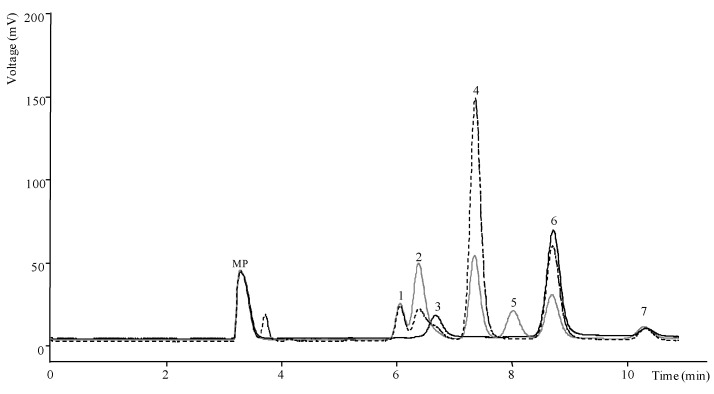
Chromatogram of sugars in cherry, plum and natural yogurts, obtained by HPLC-RI: 1—fructose; 2—glucose; 3—galactose; 4—sucrose; 5—maltose; 6—lactose; 7—raffinose (IS); MP: mobile phase.

Sucrose, a non reducing disaccharide with a α-1,2 glycosidic bound, was also present in all the studied samples, except in natural yogurt as can be observed in Figure 2. This yogurt, as expected due to the absence of fruits, presented only galactose and lactose (main sugars in milk and derivatives), and the lowest total sugars content (4.71 g/100 g). Galactose was not detected in plump yogurt but, otherwise, maltose (disaccharide with α-1,2 glycosidic bound between glucoses) was only found in this sample (Figure 2).

The highest level of total sugars was obtained in cherry yogurt (16.63 g/100 g; Table 3), which also revealed the highest value of fructose (2.02 g/100 g), and one of the highest values of sucrose and lactose (Table 3; Figure 2). Comparing yogurts with blackberry pieces is notable that blackberry “antioxidant” had higher total sugars content (14.90 g/100 g) than blackberry sample (10.04 g/100 g); the same was observed for berry yogurt “antioxidant” that showed higher levels than berry yogurt (10.41 and 7.73 g/100 g, respectively). Nevertheless, cherry yogurt “antioxidant” gave lower sugars concentration than cherry yogurt.

It should be highlighted that the values obtained by HPLC-RI analysis were similar to the ones available in the label of each yogurt (Table 3). Tocopherols are also important antioxidants in human diet, acting as free radical scavengers and preventing cellular damage through lipid peroxidation inhibition [27,28]. These compounds were determined and the results are given in Table 4. At least three of the four isoforms were present in all the samples. Blackberry yogurt with “antioxidant” mention in the label showed the highest value of total and individual tocopherols (0.29 mg/100 g, 0.02 mg/100 g, 0.29 mg/100 g and 0.34 mg/100 g for α-, β- γ-, and δ-isoforms, respectively). However, peach yogurt presented also high α-tocopherol content (0.31 mg/100 g). Otherwise, yogurt with pineapple pieces, strawberry-kiwi pieces and control (natural yogurt) gave the lowest concentration of tocopherols, without statistical significant (*p* < 0.05) differences (0.03–0.06 mg/100 g). β-tocopherol was quantified only in blackberry yogurts, but in low amounts.

**Table 4:** Tocopherols composition of the studied yogurts obtained by chromatographic analysis (mean ± SD).

	α-tocopherol	β-tocopherol	γ-tocopherol	δ-tocopherol	Total tocopherols
Berries	0.08 ± 0.03 ^c^	tr	0.06 ± 0.01 ^d^	0.06 ± 0.01 ^e^	0.20 ± 0.05 ^e^
Berries “*antioxidant*” ^a^	0.10 ± 0.01 ^c^	tr	0.11 ± 0.00 ^c^	0.10 ± 0.0 ^d^	0.31 ± 0.00 ^c^
Blackberry	0.14 ± 0.01 ^b^	0.01 ± 0.00 ^b^	0.11 ± 0.00 ^c^	0.12 ± 0.0 ^c^	0.38 ± 0.01 ^b^
Blackberry “*antioxidant*” ^a^	0.29 ± 0.03 ^a^	0.02 ± 0.00 ^a^	0.29 ± 0.01 ^a^	0.34 ± 0.00 ^a^	0.94 ± 0.02 ^a^
Cherry	0.02 ± 0.00 ^def^	nd	0.10 ± 0.01 ^c^	0.01 ± 0.00 ^h^	0.13 ± 0.01 ^f^
Cherry Burlat “*antioxidant*” ^a^	0.03 ± 0.00 ^def^	nd	0.22 ± 0.02 ^b^	0.02 ± 0.00 ^gh^	0.27 ± 0.02 ^d^
Cherry Griotte “*antioxidant*” ^a^	0.03 ± 0.01 ^de^	tr	tr	0.07 ± 0.00 ^e^	0.10 ± 0.00 ^fg^
Mango	0.09 ± 0.01 ^c^	nd	tr	0.02 ± 0.00 ^gh^	0.11 ± 0.01 ^fg^
Peach	0.31 ± 0.02 ^a^	nd	tr	0.02 ± 0.00 ^gh^	0.32 ± 0.01 ^c^
Pineapple	tr	nd	0.01 ± 0.00 ^e^	0.02 ± 0.00 ^gh^	0.03 ± 0.00 ^i^
Plum	0.05 ± 0.00 ^d^	nd	tr	0.03 ± 0.00 ^fg^	0.08 ± 0.00 ^gh^
Raspberry	0.03 ± 0.00 ^def^	nd	0.11 ± 0.04 ^c^	0.17 ± 0.01 ^b^	0.31 ± 0.01 ^c^
Strawberry-kiwi	0.02 ± 0.00 ^ef^	nd	tr	0.01 ± 0.00 ^h^	0.03 ± 0.00 ^i^
Natural (control)	0.02 ± 0.00 ^ef^	nd	tr	0.04 ± 0.00 ^f^	0.06 ± 0.01 ^ih^

^a^ These samples contain the mention “antioxidant” in the label. nd—not detected; tr—traces (below the LOQ). The results are expressed in mg/100 g fresh weight. In each column different letters (from a to h) mean statistical significant differences (“a” being the highest value) between species (*p* < 0.05).

**Figure 3:**
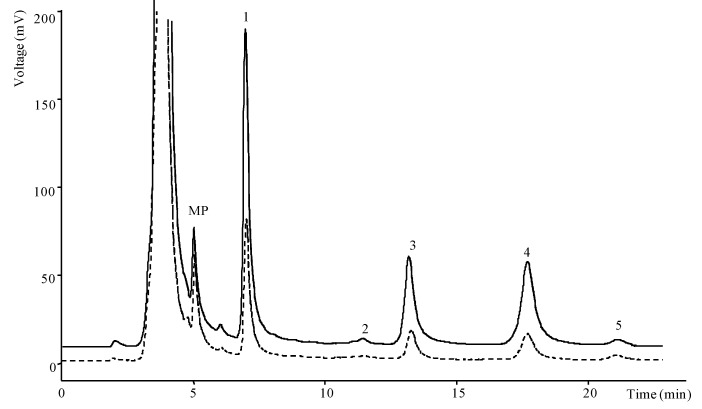
Chromatogram of tocopherols in 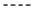 blackberry and 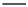 blackberry “antioxidant” yogurts, obtained by HPLC-fluorescence: 1—α-tocopherol, 2—β-tocopherol, 3—γ-tocopherol, 4—δ-tocopherols and 5—tocol (IS); MP: mobile phase.

Regarding tocopherols, the information “antioxidant” mentioned in the label seemed to be relevant, because blackberry yogurt “antioxidant” had higher values than blackberry yogurt (Figure 3; total tocopherols 0.94 mg/100 g and 0.38 mg/100 g, respectively, Table 4). Berry yogurt “antioxidant” also showed higher content than berry yogurt (except in α- and β-tocopherol) (total tocopherols 0.31 mg/100 g and 0.20 mg/100 g, respectively); the same was observed for both cherry yogurts “antioxidant” (Burlat and Griotte) that gave higher tocopherol values than cherry yogurt. Furthermore, fruit mixtures (such is the case of yogurts with pieces of berries: blackberry, strawberry and raspberry) did not show higher tocopherols content than yogurts with only one kind of fruit (for example, blackberry or raspberry yogurts). The same was concluded for strawberry-kiwi yogurt (two fruits) that gave low tocopherols content (0.03 mg/100 g).

## 4. Conclusions

Berry yogurt showed the highest phenolics (6.91 mg GAE/g extract) and flavonoids (2.98 mg CE/g extract) content, and DPPH scavenging activity (EC_50_ = 11.95 mg/mL); pineapple yogurt revealed the highest reducing power (EC_50_ = 1.74 mg/mL); blackberry yogurt gave the highest β-carotene bleaching inhibition (EC_50_ = 1.37 mg/mL); blackberry “antioxidant” yogurt showed the highest tocopherols content (0.94 mg/100 g) and cherry yogurt revealed the highest sugars content (16.63 g/100 g).

The mention of “antioxidant” in yogurts label proved to be relevant for tocopherols content (berries, blackberries and cherry yogurts showed higher levels than the corresponding “antioxidant” samples), sugars content (unless for cherry yogurts), DPPH scavenging activity and reducing power (except in the case of berry yogurts).

In general, no synergisms were observed for yogurts prepared with pieces of different fruits (berry yogurt did not show higher antioxidant properties or antioxidants content than blackberry or raspberry samples). Nevertheless, the addition of fruit pieces (natural additives) to yogurt seemed to be favorable for antioxidant contents (natural yogurt revealed low sugars—4.71 g/100 g- and tocopherols—0.06 mg/100 g-content and did not present flavonoids), increasing the protection of the consumer against diseases related to free radicals and oxidative stress. Nonetheless, it should be highlighted that other antioxidants present in the samples and not determined in the present study, such as colors, could be also responsible for the antioxidant properties observed in the different yogurts.
